# Factors Associated with Elevated Tumor Necrosis Factor-α in Aqueous Humor of Patients with Open-Angle Glaucoma

**DOI:** 10.3390/jcm11175232

**Published:** 2022-09-05

**Authors:** Younhea Jung, Kyoung Ohn, Heejong Shin, Si Eun Oh, Chan Kee Park, Hae-Young Lopilly Park

**Affiliations:** 1 Department of Ophthalmology, Yeouido St. Mary’s Hospital, College of Medicine, The Catholic University of Korea, Seoul 07345, Korea; 2 Department of Ophthalmology, Seoul St. Mary’s Hospital, College of Medicine, The Catholic University of Korea, Seoul 06591, Korea

**Keywords:** tumor necrosis factor-alpha, open angle glaucoma, normal tension glaucoma, intraocular pressure, blood pressure, vascular factor

## Abstract

Tumor necrosis factor-alpha (TNF-α) is an important modulator of neuroinflammation, secreted from activated glial cells in response to intraocular stress. The purpose of this study was to investigate the clinical factors associated with elevated TNF-α and its level in aqueous humor of patients with open-angle glaucoma (OAG). Aqueous humor was collected from 73 OAG eyes, and TNF-α level was analyzed using the singleplex bead immunoassay method. Patients were divided into TNF-α-positive and TNF-α-negative groups according to the TNF-α level of 10 pg/mL, and baseline clinical characteristics were compared. The TNF-α-positive group showed higher baseline IOP, greater IOP fluctuation, and higher systolic blood pressure than the TNF-α-negative group (*p* = 0.007, *p* < 0.001, and *p* = 0.009, respectively). In the multivariate logistic regression analysis, IOP fluctuation (*p* = 0.037) and systolic blood pressure (*p* = 0.016) were all independently associated with positive TNF-α level. In normal-tension glaucoma (NTG) patients, presence of central scotoma (*p* = 0.029) was significantly associated with positive TNF-α level. In conclusion, positive TNF-α level in OAG patients was associated with greater IOP fluctuation and higher systolic blood pressure. In NTG patients, positive TNF-α level was associated with the presence of central scotoma. IOP factors and vascular factors, including blood pressure and presence of central scotoma, may indicate glaucoma pathogenesis related to TNF-α elevation in OAG patients.

## 1. Introduction

High intraocular pressure is the most significant risk factor in glaucoma, and current therapies aim to lower intraocular pressure; however, despite successful lowering of intraocular pressure, many patients still experience disease progression [1]. In this regard, there is a growing body of evidence on the role of neuroinflammation in glaucoma [2,3,4,5,6]. During this process, resident glia and monocyte-derived cells are activated in response to persistent injury, triggering a cascade of events causing neuronal damage. Evidence of neuroinflammation, such as glial cell activation and upregulation of proinflammatory cytokines, has been confirmed in glaucoma [4,5,6].

TNF-α is an important modulator of neuroinflammation, synthesized primarily by activated monocytes due to tissue ischemia or damage [7,8]. TNF-α has neurotoxic effects and is upregulated in neurodegenerative diseases including Alzheimer’s disease, Parkinson’s disease, multiple sclerosis, and ischemic brain injuries [9]. In the eye, TNF-α is secreted from activated macrophages, astrocytes, and microglial cells in response to intraocular stress such as high pressure or ischemia, resulting in cellular apoptosis [6,8,10,11].

Elevated TNF-α levels have been previously reported in glaucoma. In glaucoma mouse models, TNF-α levels were elevated in the retina with retinal ganglion cell loss [7]. In glaucoma patients, TNF-α levels were increased in the optic nerve and the retina in glaucomatous eyes [11,12]. Furthermore, TNF-α levels were elevated in aqueous humor of patients with open-angle glaucoma (OAG) compared to normal subjects in a few studies [8,13].

Since TNF-α is secreted from activated glial cells in response to intraocular stress such as high pressure or ischemia [8,10,11], glaucoma patients with elevated TNF-α levels may show different clinical characteristics. However, the relationship between TNF-α levels and clinical characteristics has not been examined. If we could find out clinical factors related to TNF-α elevation in OAG patients, there is a possibility to use them as a biomarker to indicate neuroinflammatory mechanisms as a part of their glaucoma pathogenesis.

Therefore, in this study, we obtained aqueous humor of patients with OAG during cataract surgery and measured the level of TNF-α. We compared the clinical characteristics between TNF-α-positive and TNF-α-negative groups and analyzed factors associated with elevated levels of TNF-α. Furthermore, we also performed subanalyses in patients with normal-tension glaucoma (NTG).

## 2. Experimental Section

This study was approved by the Institutional Review Board of the Seoul St. Mary’s Hospital (KC11SISI0941) and adhered to all relevant tenets of the Declaration of Helsinki. We prospectively recruited eligible patients who were scheduled for cataract surgery and who were willing to participate. All patients provided written informed consent.

### 2.1. Subjects

Our study consisted of 80 eyes of 80 OAG patients who underwent cataract surgery using phacoemulsification at the Seoul St. Mary’s Hospital by one surgeon between July 2017 and June 2020. Patients with angle-closure or secondary etiology of glaucoma, such as uveitic glaucoma and pseudoexfoliative glaucoma, were excluded from the study. Those with any retinal disease—including diabetic or hypertensive retinopathy, history of eye trauma or previous intraocular surgery, history of refractive surgery, and any optic nerve disease or history of neurological disease that may affect the visual field—were excluded from the study.

POAG was defined as the presence of an open angle on gonioscopy, a glaucomatous optic disc (diffuse or localized rim thinning, notch in the rim, or a vertical cup-to-disc ratio of ≥0.2 compared to the other eye), visual field defects consistent with glaucoma (a cluster of ≥3 non-edge points in pattern deviation plot with a probability of <5%, with at least one of these points having a probability of <1%), a pattern standard deviation with a *p*-value <5%, or a glaucoma hemifield test result consistently outside normal limits on two preoperative visual field tests as confirmed by two glaucoma specialists (YJ and HP). Patients with normal range IOP at serial measurements at baseline were considered NTG before initiation of hypotensive medication.

Those who underwent uneventful cataract surgery were included in the study. Preoperative examination included biometry for intraocular lens calculation and blood testing for standard local surgery. All study subjects’ medical history and medical records were reviewed, including systemic symptoms such as migraine and cold extremities. All patients underwent a comprehensive ophthalmic examination, including measurement of visual acuity, intraocular pressure, slit lamp examination, measurement of central corneal thickness by ultrasound pachymetry (Tomey Corp., Nagoya, Japan), measurement of axial length with ocular biometry (IOL master; Carl Zeiss Meditec, Dublin, CA, USA), dilated stereoscopic disc photo (Canon, Tokyo, Japan), retinal nerve fiber layer (RNFL) optical coherence tomography (OCT; Cirrus, Carl Zeiss Meditec), standard automated perimetry using Swedish interactive threshold algorithm 24-2 (Humphrey; Carl Zeiss Meditec), and blood pressure. All patients were followed up with every 1–3 months for IOP measurement and optic disc evaluation. Stereoscopic disc photography, OCT, and visual field examinations were performed every year. Baseline IOP was measurement before initiation of glaucoma medication. Mean follow-up IOP was calculated as the average of all IOP measurements, and IOP fluctuation was calculated as the difference between the lowest and highest IOP during the entire follow-up period. All disc hemorrhages (DHs) detected on color disc and fundus photography during follow-up were recorded. A DH was defined as an isolated flame-shaped or splinter-like hemorrhage on the optic disc or in the parapapillary area, extending to the disc border. Alternative causes of hemorrhage (e.g., ischemic optic neuropathy, papillitis, retinal vein occlusion, diabetic retinopathy, and posterior vitreous detachment) were diagnostically excluded. Eyes with a glaucomatous visual field defect in either or both hemifields within 24 points of a central 10° of fixation, and with no visual field abnormality in the nasal periphery outside 10° of fixation, were considered to have isolated central scotoma. The criteria for central scotoma were the presence of three or more points with *p* < 5%, one of which was *p* < 1%, among 12 points on the pattern deviation plot.

### 2.2. Aqueous Humor Sample

Aqueous humor sample was collected using a standard sterilization procedure. The study protocol was approved by the institutional review board of the Seoul St. Mary’s Hospital. Prior to the beginning of cataract surgery, 50–100 μL of aqueous humor sample was collected from each eye using a 30 gauge syringe. Caution was taken to avoid inadvertent touching of intraocular tissues to prevent contamination of aqueous sample with blood. Aqueous samples were then immediately stored at −80 °C.

### 2.3. TNF-α Analysis

Samples were not diluted and processed for TNF-α analysis using singleplex bead immunoassay (Invitrogen Corporation, Waltham, MA, USA). Standards were reconstituted and serially diluted, following the manufacturer’s instructions. 25 μL of bead and wash solutions were added to each well of a 96-well microtiter plate and incubated for 30 s. Afterwards, supernatant was aspirated with a vacuum manifold. Then 50 μL of incubation buffer was added to each well, followed by 50 μL of assay diluent, and 50 μL of the aqueous humor sample. Standards were added to the designated wells and the plate was incubated for 2 h at room temperature on an orbital shaker at 250–500 rpm. After incubation period, the liquid was removed, and the excess beads were washed off. Then, detector antibody (100 μL) was added to each well and incubated on an orbital shaker for 1 h at room temperature.

After removing excess detector antibodies, substrate complex (S-RPE, streptavidin R-phycoerythrin) was added, and the fluorescence was measured using the Luminex 100TM IS fluoroanalyzer (Luminex Inc., Austin, TX, USA) after 30 min of incubation at room temperature. For each reaction, both positive and negative controls were included on the microtiter plate. As a positive control, a known concentration of human recombinant TNF-α was included in each run to ensure a proper assay.

### 2.4. Analysis of TNF-α Levels

Using standard concentration against standard fluorescence, a graphical plot was constructed. The curve fitting linear regression algorithm was performed with GraphPad InStat data analysis software (GraphPad, San Diego, CA, USA). The concentration of TNF-α level was extracted from the standard curve and expressed as pg/mL. Patients with a TNF-α level of less than 10 pg/mL were considered as the negative group which was the limit of detection. Patients with TNF-α level greater than 10 pg/mL were classified as the TNF-α-positive group based on a previous report that intra-assay coefficient of variation increases below that range [14].

### 2.5. Statistical Analysis

The baseline characteristics of the study subjects were compared using the Student’s *t*-test for continuous variables and chi-square test for categorical variables. Data are presented as mean ± standard deviation for continuous variables and number (%) for categorical variables. Univariate and multivariate logistic regression analyses were used to identify factors associated with the presence of TNF-α in aqueous humor of glaucoma patients. In addition, univariate and multivariate linear regression analyses were used to identify factors associated with the level of TNF-α. Independent variables were age, sex, history of migraine, history of cold extremities, axial length, central corneal thickness, baseline IOP, mean follow-up IOP, IOP fluctuation, baseline average peripapillary RNFL (pRNFL) thickness, baseline average macular ganglion cell-inner plexiform layer (mGCIPL) thickness, baseline mean deviation (MD), and pattern standard deviation (PSD) on standard automated perimetry, presence of DH, presence of central scotoma, and systolic and diastolic blood pressure. A subanalysis including only those with NTG was also performed.

A *p*-value of <0.05 was considered statistically significant. All statistical analyses were performed with SPSS for Windows (ver 24.0; SPSS Inc., Chicago, IL, USA).

## 3. Results

A total of 80 eyes of 80 OAG patients undergoing phacoemulsification were enrolled in the study. Of 80 eyes, 7 (8.75%) were excluded because of small sample amount. Among the remaining 73 OAG eyes, 31 eyes were TNF-α positive, whereas 42 eyes were TNF-α negative. Baseline clinical characteristics are listed in Table 1. The baseline characteristics between TNF-α-positive and TNF-α-negative groups were similar in terms of age, history of migraine, history of cold extremities, axial length, central corneal thickness, mean follow-up IOP, baseline average pRNFL thickness, baseline average mGCIPL thickness, baseline MD and PSD, presence of DH, and presence of central scotoma. The TNF-α-positive group showed higher baseline IOP (19.94 ± 8.44 mmHg) than TNF-α-negative group (15.00 ± 6.61 mmHg, *p* = 0.007). IOP fluctuation was greater in TNF-α-positive group (8.38 ± 6.29 mmHg) than TNF-α-negative group (4.36 ± 2.21 mmHg, *p* < 0.001). Systolic blood pressure was significantly higher in TNF-α-positive group (136.55 ± 15.30 mmHg) compared to TNF-α-negative group (128.37 ± 10.24 mmHg, *p* = 0.009).

We used logistic regression analysis to identify factors associated with positive TNF-α levels in aqueous humor (Table 2). Baseline IOP, IOP fluctuation, and systolic blood pressure were significantly associated with positive TNF-α level in the univariate analysis. In the multivariate logistic regression analysis, IOP fluctuation (HR = 1.231, 95% CI = 1.012–1.498; *p* = 0.037), and systolic blood pressure (HR = 1.060, 95% CI = 1.011–1.112; *p* = 0.016) were all independently associated with positive TNF-α levels.

Furthermore, to identify factors associated with TNF-α level in aqueous humor, we used linear regression analysis. TNF-α level was significantly associated with baseline IOP, mean follow-up IOP, and IOP fluctuation in univariate analysis (Table 3). In the multivariate regression analysis, mean follow-up IOP (β = 6.557, 95% CI = 2.336–10.777) was significantly associated with TNF-α level, and IOP fluctuation (β = 3.197, 95% CI = −0.021–6.415) was marginally associated with TNF-α level.

A subanalysis of those with NTG was performed. In NTG patients, baseline IOP was significantly greater in the TNF-α-positive group (15.63 ± 2.41 mmHg) compared with TNF-α-negative group (13.19 ± 2.91 mmHg, *p* = 0.005). The TNF-α-positive group included significantly greater percentage of patients with DH (*p* = 0.002) and central scotoma (*p* = 0.006, Table 4). To identify factors associated with positive TNF-α levels in NTG glaucoma patients, logistic regression analysis was performed (Table 5). Presence of DH (HR = 2.042, 95% CI = 0.310–5.222; *p* = 0.006) and presence of central scotoma (HR = 14.250, 95% CI = 2.069–98.140; *p* = 0.007) were associated with positive TNF-α level in univariate analyses. In multivariate analysis, the presence of central scotoma (HR = 7.532, 95% CI = 1.235–12.448; *p* = 0.029) was significantly associated with positive TNF-α level in the aqueous humor of NTG patients.

## 4. Discussion

In this study, we compared the clinical characteristics between TNF-α-positive and TNF-α-negative POAG patients and analyzed factors associated with elevated TNF-α level. We found that the TNF-α-positive group had higher baseline IOP, greater IOP fluctuation, and higher systolic blood pressure. In addition, a positive TNF-α level was associated with greater IOP fluctuation and higher systolic blood pressure. In the subanalysis including only NTG patients, the TNF-α-positive group showed higher baseline IOP, more frequent DH, and more frequent central scotoma. In NTG patients, positive TNF-α level was associated with the presence of central scotoma.

TNF-α is a critical modulator of neuroinflammatory response in glaucomatous neurodegeneration [6]. Elevated TNF-α signaling potentiates neuroinflammation via various pathways such as increasing the expression of Ca^2+^ permeable α-amino-3-hydroxy-5-methyl-isoxazolepropionic acid receptors and N-methyl-D-aspartate receptors and reducing the expression of inhibitory GABA receptors [6]. Thus, it enhances neuronal excitotoxicity, thereby promoting RGC loss [6,11].

Elevation of TNF-α protein, TNF-α gene expression in glial cells, and TNF-α receptor1 in retinal ganglion cells in glaucomatous eyes have been reported in previous studies [7,8,15]. In an animal study, high intraocular pressure caused rapid upregulation of TNF-α, resulting in microglial activation and sequential loss of retinal ganglion cells. Intravitreal TNF-α injection without intraocular elevation resulted in similar effects suggesting the essential role of TNF-α in glaucomatous damage [7].

Previous studies have reported elevated TNF-α levels in the aqueous humor of POAG patients. In a clinical study using an enzyme-linked immunosorbent assay, TNF-α was detected more frequently in glaucoma patients (17.8%) compared to cataract patients (5.0%) [8]. However, in their study, the TNF-α level was not associated with intraocular pressure or glaucoma severity. This may be related with different methods of measuring TNF-α. This may also partly explain the relatively low percentage of TNF-α-positive cases (10.7% in NTG, 13.7% in POAG, and 29.6% in exfoliation glaucoma) in their study. TNF-α levels can be measured even at low levels using the singleplex bead immunoassay, which is more sensitive. In a previous study using the singleplex bead immunoassay, average level of TNF-α was significantly increased in glaucomatous eyes compared to normal controls [13].

In our study, we aimed to find out associated factors to TNF-α elevation in glaucoma patients. This may reveal glaucoma patients who may have neuroinflammation and TNF-α related cell deaths as a pathogenesis of glaucomatous damage. TNF-α-positive was associated with high IOP, high IOP fluctuation, and high systolic blood pressure. These factors are well known risk factors for glaucoma progression [16,17,18,19,20]. The source of TNF-α in glaucoma is thought to be chronically reactive glial cells after injury such as high intraocular pressure [5], acute ischemia, and excitotoxicity [21,22]. These ocular and systemic risk factors may have caused glial cell activation in the eye increasing TNF-α levels and contributing to neuroinflammation. In this regard, patients with these risk factors may be associated with further glaucoma progression despite well-controlled IOP. Elevated IOP and IOP fluctuation have been reported to show glial cell activation in glaucoma animal models. Activated glial cells are an important source of TNF-α. Therefore, we confirmed this in human samples and elevated IOP and IOP fluctuation was associated with the expression of TNF-α in OAG patients [23,24].

Of note, in our subanalysis including only NTG patients, DH and central scotomas were associated with positive TNF-α level. Only central scotoma was statistically significant in the multivariate analysis. Central scotoma has been associated with vascular instability and increased risk of progression especially in NTG patients [25,26,27,28]. Glaucoma patients with central scotoma have been shown to have lower vascular density and greater fluctuation in vascular density as measured by OCT angiography [26]. Central visual field deterioration was also associated with autonomic dysfunction reflecting vascular dysregulation in these patients [25]. In patients with NTG and with central scotoma, vascular instability may contribute to glial cell activation promoting release of TNF-α. Recently, we investigated how blood pressure instability could contribute to retinal ganglion cell damage in glaucoma animal models [29]. Glial cell activation and TNF-α mediated cell death was a contributing mechanism in this glaucoma model. We confirmed that OAG patients, especially NTG patients, had an association with elevated TNF-α with central scotoma and/or DH which could be findings indicating vascular instability.

Glaucoma patients with vascular features present with central scotoma and progression in the central visual field have been linked to vascular features, including DH, autonomic dysfunction, migraines, orthostatic hypotension, and Raynaud’s phenomenon [25,30]. Therefore, vascular instability could be a contributing factor associated with TNF-α elevation that may cause glaucoma pathogenesis related to TNF-α.

## 5. Conclusions

In conclusion, this study demonstrated that POAG patients with positive TNF-α levels showed higher baseline IOP, greater IOP fluctuation, and higher systolic blood pressure. In the subanalysis including only NTG patients, those with positive TNF-α levels were associated with the presence of central scotoma. TNF-α levels are associated with IOP and vascular factors, indicating the potential role of TNF-α in glaucoma.

## Figures and Tables

**Table 1:** Comparison of baseline clinical characteristics between TNF-α-positive and TNF-α-negative open-angle glaucoma patients.

Variables	TNF-Positive Group (n = 31)	TNF-Negative Group (n = 42)	*p*-Value
Age at diagnosis (y)	65.00 ± 16.31	66.90 ± 9.25	0.526 *
Female, no. (%)	15 (48.4)	21 (50.0)	0.540 ^†^
History of migraine, no. (%)	1 (3.3)	4 (9.5)	0.301 ^†^
History of cold extremities, no. (%)	2 (6.7)	3 (7.1)	0.658 ^†^
Axial length (mm)	24.48 ± 1.53	24.15 ± 1.54	0.380 *
Central corneal thickness (μm)	544.00 ± 45.18	532.66 ± 32.23	0.282 *
Baseline IOP (mmHg)	19.94 ± 8.44	15.00 ± 6.61	0.007 *
Mean follow-up IOP (mmHg)	14.61 ± 4.28	13.29 ± 3.04	0.128 *
IOP fluctuation (mmHg)	8.38 ± 6.29	4.36 ± 2.21	<0.001 *
Baseline average pRNFL thickness (μm)	68.94 ± 13.23	67.19 ± 23.59	0.712 *
Baseline average mGCIPL thickness (μm)	57.67 ± 24.73	50.81 ± 31.42	0.317 *
Baseline MD of SAP (dB)	−10.27 ± 9.40	−6.90 ± 6.33	0.071 *
Baseline PSD of SAP (dB)	5.99 ± 4.14	5.24 ± 4.21	0.452 *
Presence of DH, no. (%)	2 (6.5)	3 (7.3)	0.632 ^†^
Presence of central scotoma, no (%)	12 (38.7)	17 (40.5)	0.537 ^†^
Systolic blood pressure (mmHg)	136.55 ± 15.30	128.37 ± 10.24	0.009 *
Diastolic blood pressure (mmHg)	77.17 ± 10.25	75.88 ± 8.26	0.561 *
TNF alpha level in aqueous humor (pg/mL)	60.24 ± 73.95	1.12 ± 2.72	<0.001 *

Data are presented as mean ± standard deviation for continuous variables and number (%) for categorical variables. * Student’s *t*-test. ^†^ Chi-square test. IOP: intraocular pressure, pRNFL: peripapillary retinal nerve fiber layer, mGCIPL: macular ganglion cell inner plexiform layer, MD: mean deviation, SAP: standard automated perimetry, PSD: pattern standard deviation, DH: disc hemorrhage, TNF: tumor necrosis factor.

**Table 2:** Factors associated with TNF-α-positive in the aqueous humor of open-angle glaucoma patients.

Variables	Univariate		Multivariate	
	HR (95% CI)	*p*-Value	HR (95% CI)	*p*-Value
Age at diagnosis (y)	0.998 (0.952–1.025)	0.522		
Female, no. (%)	0.938 (0.370–2.372)	0.892		
History of Migraine, no. (%)	3.053 (0.324–28.788)	0.330		
History of cold extremities, no. (%)	1.077 (0.169–6.876)	0.938		
Axial length (mm)	1.148 (0.845–1.561)	0.376		
Central corneal thickness (μm)	1.008 (0.994–1.023)	0.279		
Baseline IOP (mmHg)	1.104 (1.017–1.197)	0.018	1.039 (0.946–1.141)	0.422
Mean follow-up IOP (mmHg)	1.112 (0.966–1.279)	0.139		
IOP fluctuation (mmHg)	1.303 (1.087–1.561)	0.004	1.231 (1.012–1.498)	0.037
Baseline average pRNFL thickness (μm)	1.005 (0.981–1.029)	0.708		
Baseline average mGCIPL thickness (μm)	1.009 (0.992–1.026)	0.314		
Baseline MD of SAP (dB)	0.946 (0.890–1.006)	0.075	0.975 (0.899–1.057)	0.542
Baseline PSD of SAP (dB)	1.045 (0.934–1.169)	0.446		
Presence of DH, no. (%)	1.145 (0.179–7.304)	0.886		
Presence of central scotoma, no (%)	1.077 (0.417–2.783)	0.879		
Systolic blood pressure (mmHg)	1.056 (1.010–1.104)	0.016	1.060 (1.011–1.112)	0.016
Diastolic blood pressure (mmHg)	1.016 (0.963–1.072)	0.555		

IOP: intraocular pressure, pRNFL: peripapillary retinal nerve fiber layer, mGCIPL: macular ganglion cell inner plexiform layer, MD: mean deviation, SAP: standard automated perimetry, PSD: pattern standard deviation, DH: disc hemorrhage.

**Table 3:** Factors associated with the level of TNF-α in the aqueous humor of open-angle glaucoma patients.

Variables	Univariate		Multivariate	
	β (95% CI)	*p*-Value	β (95% CI)	*p*-Value
Age at diagnosis (y)	−1.022 (−2.050–0.006)	0.051	0.102 (−1.125–1.329)	0.869
Female, no. (%)	0.938 (0.370–2.372)	0.892		
History of Migraine, no. (%)	3.053 (0.324–28.788)	0.330		
History of cold extremities, no. (%)	1.077 (0.169–6.876)	0.938		
Axial length (mm)	7.185 (−1.315–15.886)	0.096	5.930 (−2.812–14.673)	0.180
Central corneal thickness (μm)	−3.033 (−6.717–0.651)	0.105		
Baseline IOP (mmHg)	1.681 (0.022–3.341)	0.047	−1.591 (−3.940–0.758)	0.181
Mean follow-up IOP (mmHg)	5.964 (2.610–9.319)	0.001	6.557 (2.336–10.777)	0.003
IOP fluctuation (mmHg)	3.278 (0.636–5.920)	0.016	3.197 (−0.021–6.415)	0.050
Baseline average pRNFL thickness (μm)	−0.132 (−0.803–0.539)	0.696		
Baseline average mGCIPL thickness (μm)	0.040 (−0.421–0.501)	0.864		
Baseline MD of SAP (dB)	−1.401 (−3.048–0.246)	0.094	−1.160 (−2.815–0.495)	0.166
Baseline PSD of SAP (dB)	−0.280 (−3.465–2.906)	0.862		
Presence of DH, no. (%)	1.145 (0.179–7.304)	0.886		
Presence of central scotoma, no (%)	1.077 (0.417–2.783)	0.879		
Systolic blood pressure (mmHg)	0.508 (−0.103–1.118)	0.243		
Diastolic blood pressure (mmHg)	0.453 (−0.440–1.347)	0.315		

IOP: intraocular pressure, pRNFL: peripapillary retinal nerve fiber layer, mGCIPL: macular ganglion cell inner plexiform layer, MD: mean deviation, SAP: standard automated perimetry, PSD: pattern standard deviation, DH: disc hemorrhage.

**Table 4:** Comparison of baseline characteristics between TNF-α-positive and TNF-α-negative normal tension glaucoma patients.

Variables	TNF-Positive Group (n = 10)	TNF-Negative Group (n = 21)	*p*-Value
Age at diagnosis (y)	70.84 ± 10.19	67.00 ± 10.56	0.228 *
Female, no. (%)	8 (80.0)	10 (47.6)	0.092 ^†^
History of Migraine, no. (%)	1 (10.0)	2 (9.5)	0.704 ^†^
History of cold extremities, no. (%)	2 (20.0)	1 (4.8)	0.237 ^†^
Axial length (mm)	24.78 ± 1.43	24.37 ± 1.82	0.423 *
Central corneal thickness (μm)	542.79 ± 40.65	539.75 ± 35.07	0.817 *
Baseline IOP (mmHg)	15.63 ± 2.41	13.19 ± 2.91	0.005 *
Mean follow-up IOP (mmHg)	13.68 ± 3.05	12.74 ± 3.09	0.317 *
IOP fluctuation (mmHg)	5.84 ± 2.89	4.34 ± 2.35	0.062 *
Baseline average pRNFL thickness (μm)	66.58 ± 9.19	66.42 ± 11.17	0.961 *
Baseline average mGCIPL thickness (μm)	50.36 ± 28.14	49.69 ± 27.95	0.937 *
Baseline MD of SAP (dB)	−8.28 ± 8.56	−8.56 ± 6.09	0.896 *
Baseline PSD of SAP (dB)	6.38 ± 4.18	6.45 ± 4.19	0.959 *
Presence of DH, no. (%)	5 (50.0)	0	0.002 ^†^
Presence of central scotoma, no (%)	6 (60.0)	2 (9.5)	0.006 ^†^
Systolic blood pressure (mmHg)	134.35 ± 11.80	128.60 ± 10.75	0.110 *
Diastolic blood pressure (mmHg)	76.35 ± 11.50	76.92 ± 8.71	0.857 *
TNF alpha level in aqueous humor (pg/mL)	62.08 ± 86.11	1.66 ± 3.27	0.001 *

Data are presented as mean ± standard deviation for continuous variables and number (%) for categorical variables. * Student’s *t*-test. ^†^ Chi-square test. IOP: intraocular pressure, pRNFL: peripapillary retinal nerve fiber layer, mGCIPL: macular ganglion cell inner plexiform layer, MD: mean deviation, SAP: standard automated perimetry, PSD: pattern standard deviation, DH: disc hemorrhage, TNF: tumor necrosis factor.

**Table 5:** Factors associated with TNF-α-positive in the aqueous humor of normal tension glaucoma patients.

Variables	Univariate		Multivariate	
	HR (95% CI)	*p*-Value	HR (95% CI)	*p*-Value
Age at diagnosis (y)	1.065 (0.978–1.161)	0.149		
Female, no. (%)	4.400 (0.749–25.842)	0.101		
History of migraine, no. (%)	0.947 (0.076–11.870)	0.947		
History of cold extremities, no. (%)	0.200 (0.016–2.527)	0.214		
Axial length (mm)	0.890 (0.500–1.583)	0.691		
Central corneal thickness (μm)	1.030 (0.816–1.301)	0.801		
Baseline IOP (mmHg)	1.318 (0.952–1.824)	0.097	1.174 (0.776–1.777)	0.448
Mean follow-up IOP (mmHg)	1.085 (0.844–1.394)	0.525		
IOP fluctuation (mmHg)	1.090 (0.774–1.534)	0.622		
Baseline average pRNFL thickness (μm)	1.034 (0.957–1.117)	0.393		
Baseline average mGCIPL thickness (μm)	1.046 (0.979–1.117)	0.181		
Baseline MD of SAP (dB)	1.161 (0.977–1.381)	0.190		
Baseline PSD of SAP (dB)	0.930 (0.759–1.139)	0.484		
Presence of DH, no. (%)	2.042 (0.310–5.222)	0.006	2.252 (0.037–6.612)	0.093
Presence of central scotoma, no (%)	14.250 (2.069–98.140)	0.007	7.532 (1.235–12.448)	0.029
Systolic blood pressure (mmHg)	1.009 (0.941–1.082)	0.802		
Diastolic blood pressure (mmHg)	0.956 (0.880–1.040)	0.296		

IOP: intraocular pressure, pRNFL: peripapillary retinal nerve fiber layer, mGCIPL: macular ganglion cell inner plexiform layer, MD: mean deviation, SAP: standard automated perimetry, PSD: pattern standard deviation, DH: disc hemorrhage.

## Data Availability

The datasets used and/or analysed during the current study are available from the corresponding author on reasonable request.
